# Forward or backward, that is the question: phospholipid trafficking by the Mla system

**DOI:** 10.1042/ETLS20220087

**Published:** 2022-12-02

**Authors:** Javier Abellon-Ruiz

**Affiliations:** Biosciences Institute, Faculty of Medical Sciences, Newcastle University, Newcastle upon Tyne NE2 4HH, U.K.

**Keywords:** asymetry, gram negative bacteria, outer membrane

## Abstract

The distinctive feature of Gram-negative bacteria is the presence of an asymmetric outer membrane (OM), which acts as a permeation barrier blocking the diffusion of noxious components such as antibiotics that could compromise cell survival. The outer membrane has an inner leaflet, mainly formed by phospholipids (PLs), and the outer leaflet, composed of molecules of lipopolysaccharide (LPS). Building this membrane is a very complex process as every OM element needs to be transported from the cytoplasm or the inner membrane and properly placed in the OM. In addition, the asymmetry needs to be maintained to guarantee the barrier function of the membrane. The presence of misplaced PLs in the outer leaflet of the OM causes increased permeability, endangering cell survival. The Mla system (maintenance of OM lipid asymmetry) has been linked to the removal of the misplaced PLs, restoring OM asymmetry. The Mla system has elements in all compartments of the cell envelope: the lipoprotein MlaA in complex with the trimeric porins OmpC/F in the OM, MlaC in the periplasmic space and an ABC transporter in the inner membrane called MlaFEDB. While genetic and structural work suggest that the Mla pathway is retrograde (PL movement from OM to IM), several groups have advocated that transport could happen in an anterograde fashion (from IM to OM). However, recent biochemical studies strongly support retrograde transport. This review provides an overview of the current knowledge of the Mla system from a structural point of view and addresses the latest biochemical findings and their impact in transport directionality.

## Introduction

**Figure 1.**
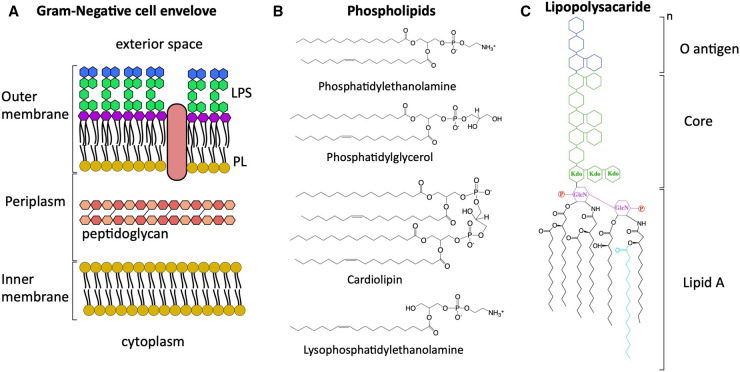
Architecture of the Gram-negative cell envelope. (**A**) Diagram showing the four elements of the Gram-negative cell envelope. LPS lipopolysaccharide, PL phospholipid. Note the outer membrane protein depicted in pale pink (**B**) Chemical structure of membrane glycerophospholipids of *Escherichia coli* and a molecule of lysophospholipid. (**C**) Diagram showing the simplified chemical structure of *E. coli* lipopolysaccharide. Kdo: 3-deoxy-D -manno-octulosonic acid, GlcN: D-glucosamine, sugars are represented as blue or green hexagons, phosphate group depicted as P in red. The acyl chain in cyan represents the palmitate transferred from a PL to the lipid A of a molecule of LPS by PagP, generating a hepta-acylated lipid A.

The Gram-negative cell envelope comprises four elements: the inner membrane (IM), the outer membrane (OM), separated by the periplasmic space that contains a thin peptidoglycan (PG) layer [1] (Figure 1A). The presence of this OM is a distinctive feature of Gram-negative bacteria and acts as a permeation barrier [2]. The OM is highly asymmetric and consists of an environment-exposed outer leaflet composed of lipopolysaccharide (LPS) and an inner leaflet of glycerophospholipids (PLs) [3,4]. Although the composition of PLs of this inner leaflet, and the IM, varies across bacterial species, this review will consider the model bacterium *E. coli* which presents three types of PLs: phosphatidylethanolamine, phosphatidylglycerol and cardiolipin [5] (Figure 1B). The OM offers protection not only against large polar molecules but also from lipophilic compounds, including antibiotics, detergents and other environmental noxious molecules [2]. The low permeability of the OM is due to the nature of LPS. Although the general structure and properties of LPS are conserved, modifications occur at the species and strain level [6,7]. The *E. coli* LPS molecule consists of lipid A (a disaccharide of glucosamine phosphorylated and substituted with saturated hydroxylated acyl chains) ligated to a core oligosaccharide (around 10 sugars), and attached to this core is the O antigen (a highly variable polymer that can have more than 100 sugars) [8] (Figure 1C). The phosphate groups of the disaccharide of glucosamine from neighbour LPS molecules interact with divalent cations (e.g. Ca^2+^ or Mg2^+^) forming a strong lateral interaction which leads to tight packing. This, together with the hydrophobic interactions of the acyl chains from the lipid A, produces a strong barrier for small hydrophobic compounds [2,9]. To overcome this barrier and import nutrients and other molecules, Gram-negative bacteria have outer membrane proteins (OMPs) in the shape of barrels composed of antiparallel β-strands (Figure 1A). There are three different types of transporters: energy-coupled, non-specific diffusion channels called porins (substrate <600 Da, e.g. OmpF and OmpC) and substrate-specific channels (up to ∼1.2 kDa) [10–12]. LPS, PLs and OMPs are synthesised in the cytoplasm or the IM and then transported to the OM, making the building of this membrane an extremely complex and coordinated process [13–18]. In addition, the integrity and asymmetry of the OM needs to be maintained to keep the barrier function in place. Remarkably, these processes are constrained by the fact that the OM is semi-permeable in both directions, and does not provide external energy from ATP or ion gradients.

The presence of PLs in the outer leaflet disrupts the asymmetric nature of the OM and represents a threat to cell survival. PLs and LPS do not mix, but form patches resulting in regions of PLs double bilayer [19,20] allowing the diffusion of harmful compounds [21]. To re-establish OM asymmetry, Gram-negative bacteria have two OMPs that modify these mislocalised PLs: PagP and PldA. PagP is a palmitoyltransferase that transfers a palmitate chain from PLs to lipid A. This reaction releases a hepta-acylated lipid A and a lysophospholipid (Figure 1B,C), the latter will have to be degraded or transported to the IM and regenerated [22,23]. PldA is an OM phospholipase which degrades surface exposed PLs or lysophospholipids, releasing fatty acids that will be transported to the cytoplasm [24,25]. It is important to stress that these two systems modify outer leaflet PLs but do not remove them.

**Figure 2.**
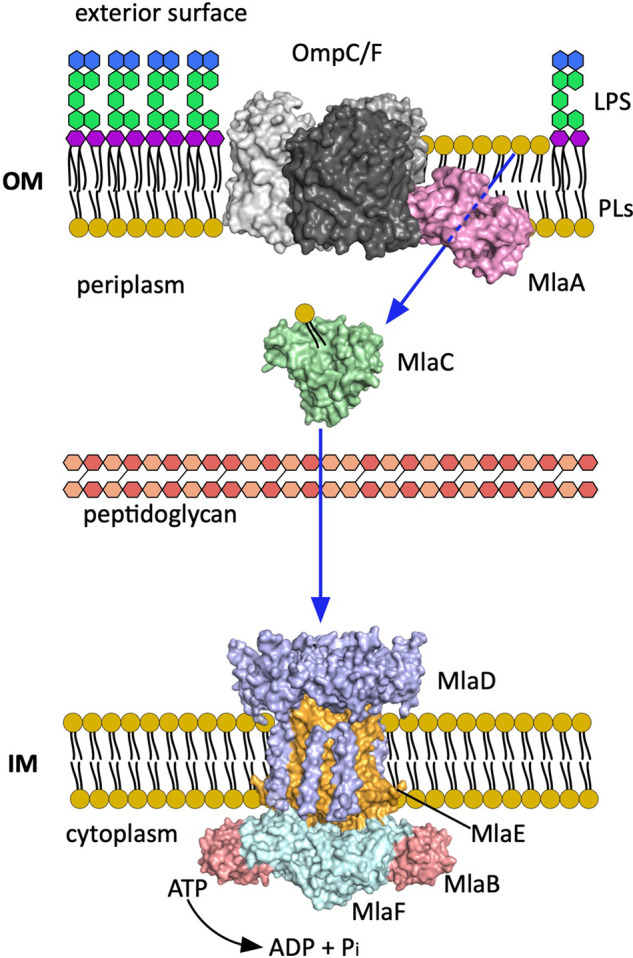
Overview of the Mla pathway. Localisation of the elements of the Mla system in the cell envelope. Each monomer of the trimeric porin OmpF/C is represented in different shades of grey. The MlaFEDB components are coloured by type of protein and not by monomers. The blue arrows indicate the most accepted PLs movement direction (retrograde). (PDB codes; MlaA–OmpF/C 5NUO [33], MlaC 5UWA [34], MlaFEDB 6XBD [35]).

In 1977 Jones and Osborn showed that PL vesicles incubated with viable cells of *Salmonella typhimurium* resulted in PLs travelling to the IM, being modified there and then equilibrated with the OM, suggesting a bidirectional mechanism of PLs transport [26]. We had to wait over 30 years until Malinverni and Silhavy discovered the first components of a PL trafficking system in *E. coli*. They described a system that maintains the asymmetry of the OM by moving PLs from the outer leaflet of the OM to the IM (retrograde transport), and they called it the Mla pathway (maintenance of lipid asymmetry) [27]. As we will see later, for some groups the transport directionality is still a subject of debate, although the retrograde mode is the most widely accepted and supported by genetic, structural and biochemical work. The Mla system has three elements: the complex MlaA–OmpC/F located in the OM, the periplasmic lipid binding soluble protein MlaC and the ABC transporter (MlaFEDB) in the IM [27,28] (Figure 2). The Mla pathway is conserved in plants and in Gram-negative bacteria [29,30]. Homologues of MlaF, MlaE, and MlaD (described as uptake lipid transporters in Actinobacteria [31] and retrograde PL transporters in plant chloroplasts [32]) led to Malinverni and Shilhavy to explore their function in *E.coli*. Their work showed that loss-of-function mutations in any of the Mla pathway genes caused cells to be sensitive to SDS-EDTA, suggesting a defective OM with increased permeability. They proposed that in the absence of a functional Mla pathway PLs are accumulating in the outer leaflet of the OM [27]. To further support this hypothesis, the overexpression of PldA restores the barrier function via hydrolysis of the PLs accumulated in the outer leaflet in the absence of a functional Mla system.

Intriguing is the gain of function phenotype of the mlaA* mutant discovered by Sutterlin and co-workers. Strains carrying this mutation (MlaA ΔAsn41–Phe42) showed an increased sensitivity to SDS-EDTA compared with null mutants of the Mla pathway [36]. This mutant caused a higher accumulation of PLs in the outer leaflet of the OM than null mutants and it was proposed to work in the reverse direction as the wild-type MlaA [36]. Although the molecular mechanism of MlaA was not clear at that point, all the functional experiments linked this pathway with the maintenance of OM asymmetry.

## The MlaA–OmpC/F complex

**Figure 3.**
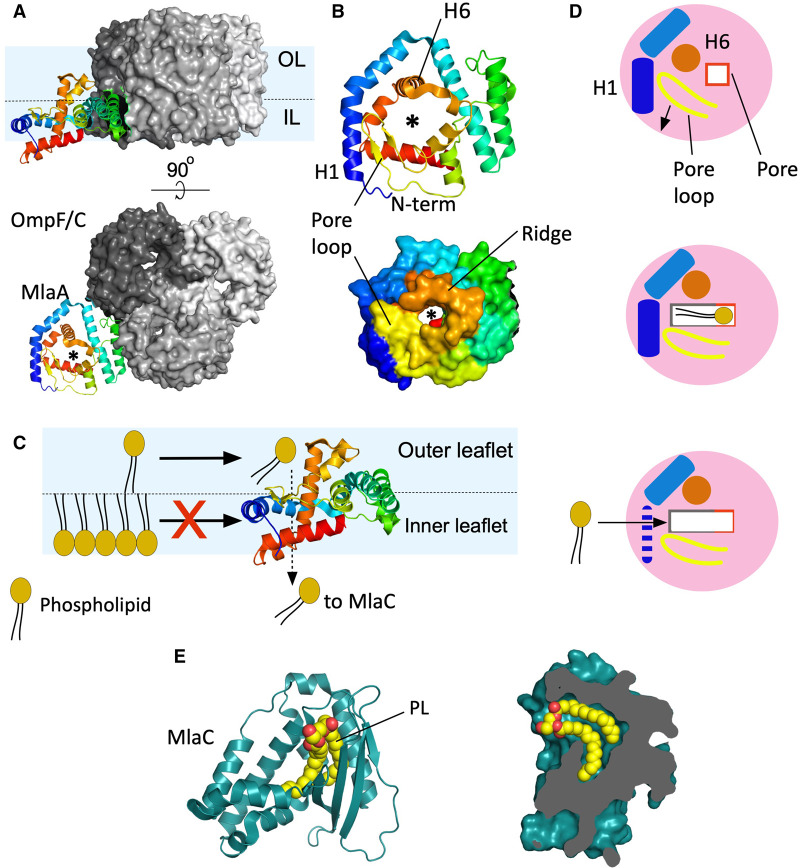
The MlaA–OmpC/F complex and MlaC. (**A**) Representation of a MlaA–OmpF/C complex from the OM plane (top panel) and viewed from the outside of the cell (bottom panel). The schematic representation of MlaA is in rainbow colouring (N-terminus; blue). The surface model of OmpF/C trimers shows each monomer in a different shade of grey. The asterisk indicates the MlaA channel. The inner leaflet (IL) and outer leaflet (OL) of the OM are separated by a dashed line in the top panel. (**B**) Exterior view of MlaA (schematic representation and surface view, top and bottom panels, respectively). The most significant structural elements have been labelled. (**C**) Diagram showing the proposed retrograde transport mechanism for MlaA viewed from the OM plane. Helices around the central channel, resembling a donut, are blocking the access of PLs from the inner leaflet into the pore while PLs located in the outer leaflet have access to the channel and are transferred to MlaC. (**D**) Schematic view of MlaA from the outside of the cell. Top panel represent the crystal structure, the hydrophilic pore is shown as a red line square. The arrow indicates the direction of the structural rearrangement needed to open up the pore. Middle panel, diagram of the open state in which the pore is enlarged and the hydrophobic region of the channel (grey line) is exposed. For clarity only helices 1 and 6 are represented and labelled. Pore loop in yellow. Bottom panel, proposed disruption of H1 in mutant mlaA* allowing direct access of PLs from the inner leaflet into the channel. (**E**) Left, schematic representation of MlaC (aquamarine-blue) with a PL bound (in yellow and red). Right, slice of a surface representation of MlaC showing the hydrophobic pocket with a PL bound.

MlaA was predicted to be a periplasmic exposed lipoprotein implicated in removing PLs from the outer leaflet of the OM. But how is a periplasmic protein gaining access to the exterior part of the cell envelope? Work of Chng and collaborators showed that MlaA forms a complex with the trimeric porins OmpC and OmpF, but only the interaction with OmpC appeared to be biologically relevant [27]. This discovery raised more questions about the MlaA molecular mechanism. How are MlaA and OmpC removing PLs from the outer leaflet of the OM? Is MlaA enabling the access of PLs into the porin channel? Is MlaA directly removing PLs? What is the function of the porin? Structural and functional work from the van den Berg laboratory offered a mechanistic explanation of MlaA function in the OM, addressing many of these questions. The crystal structures showed that MlaA is a ring-shaped α-helical protein almost entirely embedded into the inner leaflet of the OM and bound in the groove of two monomers of OmpC/F (Figure 3A) [33] (for a detailed biochemical study of the interaction surface porin-MlaA consult [37] from Chng's laboratory). Structures for MlaA–OmpF and MlaA–OmpC are virtually identical, raising questions why only MlaA–OmpC, and not MlaA–OmpF, would be active [27]. Gratifyingly, MlaA has a central amphipathic channel whose size is constrained by helix 6 (H6) and a loop (referred here as pore loop). This channel has a semi-circular ridge with its top end located at the interface of the outer leaflet (Figure 3B). Molecular dynamics simulations showed that the polar headgroups of PLs misplaced in the outer leaflet interact with the ridge of MlaA and move downwards into the amphipathic channel which causes tilting of the acyl chains (Figure 3C). Abellón-Ruiz et al. proposed that to allow the diffusion of PLs, MlaA needs to undergo a conformational change. This could be spontaneous or as a result of the binding of MlaC to MlaA to accept the PL. This change would involve a displacement of the pore loop and helix 6, exposing a hydrophobic region of the channel wide enough to allocate the acyl chains (Figure 3C,D). The authors supported this hypothesis with functional analysis, by introducing two cysteines in opposite sides of the pore (one in the loop and the other in helix 6), ‘locking’ the structure and preventing the opening of the channel. As a result, MlaA function is lost and the strain is more sensitive to exogenous stressors compared with WT cells. Of note, single cysteine mutations do not cause a loss of function. The addition of a reducing agent recovers the wild-type phenotype, probably by disulfide bond reduction, allowing the conformational change to happen. Biochemical work from Chng's laboratory further supports the key role of this loop for the MlaA function [37]. Another interesting feature of MlaA structure is that all the α-helices, except H6, run parallel to the OM and as a consequence they act as a barrier preventing the access of PLs from the inner leaflet to the pore (Figure 3C). This disposition offers a possible explanation for the mlaA* mutant; the helix 1 deletion likely breaks the barrier, allowing PLs from the inner leaflet to reach the MlaA channel and move into the outer leaflet, driven by the difference of concentration of the PLs in both leaflets (Figure 3D). Thus, the structures explain how MlaA selectively accepts PLs from the outer leaflet while blocking access of inner leaflet PLs. The structures also suggest that the role of the porin is to stably position MlaA at the right depth in the OM (Figure 3A). It might be very informative to obtain MlaA structures via cryo-EM in nanodiscs to possibly observe PLs in the channel and the proposed conformational changes.

## Mlac

The transfer of PLs between the Mla pathway components located in the OM and the IM is carried out by MlaC, a periplasmic soluble protein. MlaC has a hydrophobic pocket which binds the acyl chains of phospholipids, leaving the polar head exposed to the media [34] (Figure 3E). This binding has a high affinity evidenced by the fact that *E. coli* MlaC (*Ec*MlaC) copurifies with bound PLs [34,38]. *Ec*MlaC binds PLs with two or four acyl chains covering the three types of PLs present in *E. coli*, but there are no structures conclusively showing a bound cardiolipin molecule to it [34,39]. Recently, Yero et al. [40] have shown a structure of MlaC (Ttg2D) from *Pseudomonas aeruginosa* with two PLs bounds. Using native mass spectrometry, they confirm that Ttg2D can accommodate four acyl chains and it can transport two PLs (heterologously or homologously) or one molecule of cardiolipin [40]. MlaC does not copurify with MlaA nor with the MlaFEDB complex but it has been reported to interact with MlaA and MlaD [34,38]. Despite many efforts to elucidate how transfer of PLs from MlaA to MlaC happens, this is still not clear. It seems reasonable to assume transfer is driven by affinity, as MlaC likely has a higher affinity for PLs than MlaA. So far, no structure of MlaA-PL has been reported, which is reasonable as MlaA is a PL diffusion channel in which a high affinity for PLs would make transport inefficient. Spontaneous transfer of PLs from MlaD to MlaC, independent of ATP hydrolysis, has been reported, which is at odds with the widely accepted retrograde transport model [38,39] . However, a more recent biochemical analysis has shown that the hydrolysis of ATP prevents the observed spontaneous transfer of PLs from MlaD to MlaC abolishing the anterograde transport [41].

## The MlaFEDB complex

**Figure 4.**
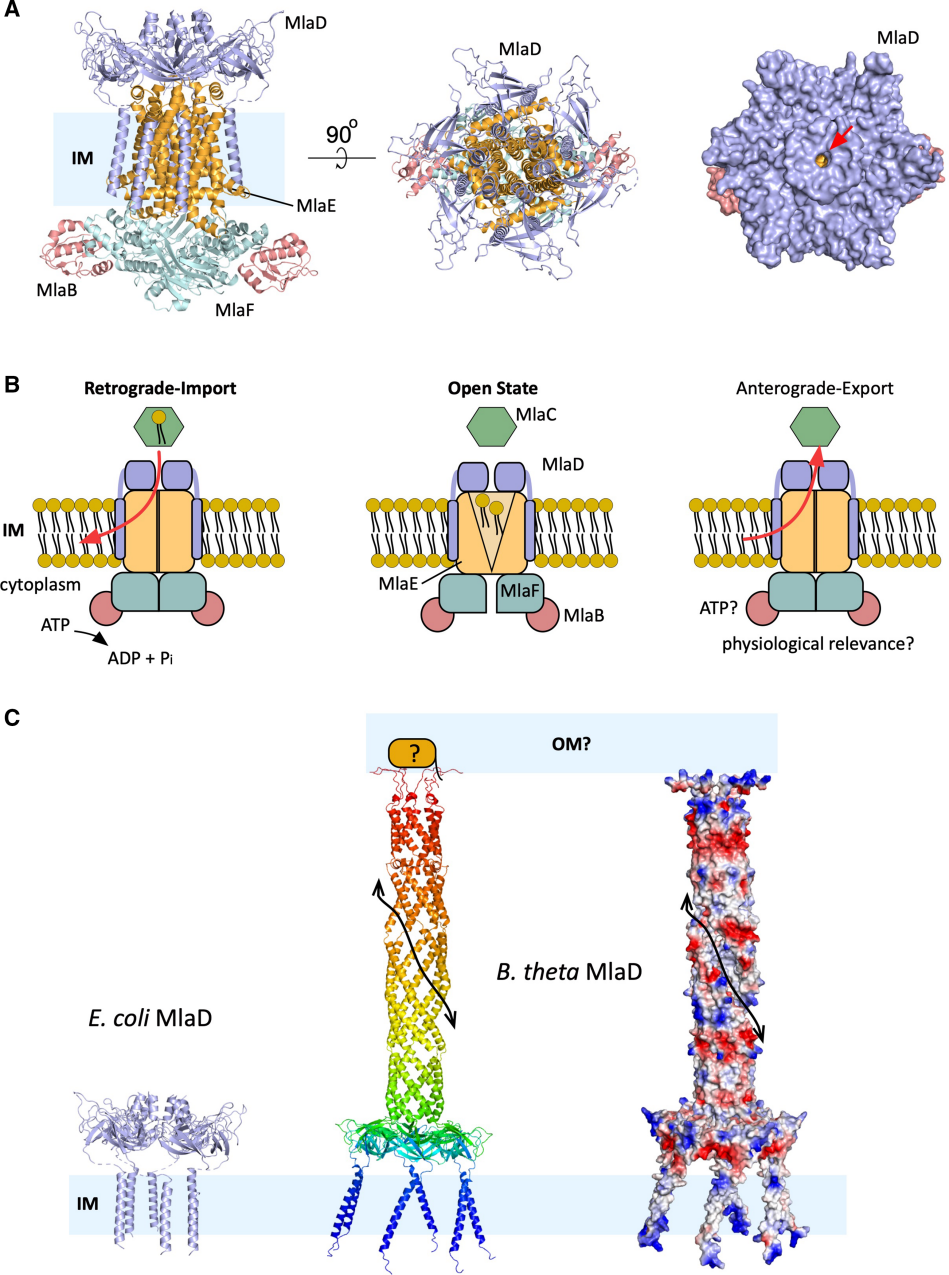
Overview of the MlaFEDB complex. (**A**) Representation of the MlaFEDB complex from the IM plane and periplasm view (left and middle panels). Surface view of the complex from the periplasm, right panel. The red arrow shows the hydrophobic channel. (**B**) Schematic diagrams showing the open complex state and the two possible transport directions. For clarity the colour code has been maintained in A and B. (**C**) Representation of *E. coli* MlaD compared with the AlphaFold model predicted for *B. theta*. The middle panel shows a rainbow coloured representation (blue, N-terminus) and the right panel shows the electrostatic surfaces (−53 kT e^−1^ to 53 kT e^−1^) of the predicted structure (negative residues in red, positive in blue). The potential interaction site with the OM and the predicted structure is shown. The possibility of an unknown associated OM lipoprotein is depicted. The black arrow shows the portion of the channel groove. For clarity, the remainder of the ABC transporter is not shown.

The IM component of the Mla pathway is the ABC (ATP-binding cassette) transporter MlaFEDB. The complex has a stoichiometry 2 : 2 : 6 : 2 and all the elements copurify when overexpressed in *E. coli* [34,42,43]. MlaE and MlaF are the core elements of the ABC transporter. These homodimers function as the transmembrane domains (TMDs) and the nucleotide-binding domains (NBDs) respectively. The accessory protein MlaB is bound to MlaF on the cytoplasmic side of the complex (Figure 4A) and has been implicated in stabilising the complex and ATP hydrolysis [43,44]. MlaD has a periplasmic region with a mammalian cell entry (MCE) domain and a transmembrane helix. The MCE domains have been implicated in lipid uptake in Gram-negative bacteria and retrograde transport of PLs in chloroplasts [32,45]. MlaD forms a ring-shaped homohexamer defining a central a hydrophobic pore that is proposed to allow PLs to move through [34,38,39]. The MlaD hexamer sits on top of MlaE and it is anchored by the six transmembrane helices [33,36,37] (Figure 4A). Recent cryo-EM structural work from different laboratories has provided further insight into this intriguing complex. The structures from *E. coli*, *Pseudomonas aeruginosa* and *Acinetobacter baumannii* show that, in the absence of ATP, MlaE adopts a V-shape open conformation to form a cavity with the wider side facing the hydrophobic channel of MlaD. Several of the structures have density in the cavity and in other regions of the complex that could correspond to PLs or bound detergent [35,46–50]. These recent studies also clarify that MlaE has five helices and enable a structure-based comparison analysis which shows that MlaE is similar to the LPS export system. But the arrangement of the transmembrane helices is different enough to consider MlaE the founding member of the type VIII group of ABC TMDs (for a more detailed discussion consult [51]). In retrograde transport, the energy released after the hydrolysis of ATP would be used to extract the PLs from MlaC and then transfer them to MlaD. After that, PLs move into the MlaE cavity and eventually are incorporated into the IM (Figure 4B).

## Transport directionality

Genetic assays strongly support retrograde transport but contrast with early biochemical results that show ATP-independent transfer of PLs from MlaD to MlaC [38,39]. However, in these experiments complexes of MlaA–OmpF/C were not present (for more details see [52]). To overcome this, Tang and collaborators mixed proteoliposomes with MlaA–OmpF or the MlaFEDB complex, adding fluorescently labelled PLs to one or the other proteoliposome population. This experiment was designed so that the transfer of PLs between vesicles could be monitored measuring the variation in fluorescence. These assays demonstrated ATP-dependent transport that was predominantly retrograde, in agreement with the genetic data [46].

In addition, recent biochemical work from Chng's laboratory shows that the hydrolysis of ATP is key to prevent the spontaneous transfer of PLs from MlaD to MlaC thus blocking anterograde transport [41]. The Trent laboratory has also reported that the Mla system has no role in anterograde lipid transport in *Acinetobacter baumannii* [53]. Most papers advocating anterograde PL transport by the Mla system ignore the structures of MlaA–OmpF/C complexes, which strongly suggest that in hypothetical anterograde transport, PLs will be delivered to the OM outer leaflet, making the cell more sensitive to noxious compounds, which would appear nonsensical. Moreover, since only one or two PLs are bound by MlaC and transferred to MlaA at a time, the efficiency of the Mla system for building the OM would seem very low. In contrast, the number of outer leaflet PLs at any time is likely low, making a one/two-at-a-time removal by MlaA reasonable. The efficiency argument becomes even stronger in light of the recent discoveries of AsmA-like proteins and their possible roles in PL transport in *E. coli*. These proteins are predicted to have structural homology to eukaryotic lipid transporters [13,18], with a domain anchored into the IM and a periplasmic domain shaped like a taco with a hydrophobic interior. This invokes a picture, analogous to LPS transport by the Lpt complex, of conveyor belts delivering PLs to the OM in a much more efficient manner than would be possible by anterograde Mla transport.

## Future perspectives

As detailed above, the structural studies on MlaA–OmpF/C show that the most likely role for the trimeric porins is placing or stabilising MlaA in the right position on the OM to create a channel between the periplasm and the outer leaflet, bypassing the inner leaflet. MlaA–OmpF and MlaA–OmpC are virtually the same [33], bringing into question what drives the differences seen in the functional and genetic assays between both complexes [28]. To answer this, it would be beneficial to understand what happens in other Pseudomonadota (formerly Proteobacteria) such as *Acinetobacter baumannii* or *Pseudomonas aeruginosa*. Both of them lack OmpF and OmpC porins but they have MlaA homologues. AlphaFold2 [54,55] predicts that their MlaA structures are very similar to *E. coli* MlaA. How are they placed into the OM in the absence of OmpF/C? These organisms possess different trimeric porins such as OprO [56] and OprP [57] in *P. aeruginosa* and DcaP [58] in *A. baumannii*. These channels have 16 beta strands (the same as OmpF/C) and it seems reasonable to assume that they could interact with MlaA. These studies could clarify the role of porins in the Mla pathway or identify new partners for MlaA (or alternatively, confirm their absence). Even more interesting would be to explore what happens in other bacteria outside the Pseudomonadota phylum. Given the implication of the phylum Bacteroidota (formerly Bacteroidetes) in human health, exploring how the Mla system works in this group of bacteria could be very informative. The genome of *Bacteroides thetaiotamicron* (*B. theta*), a prominent gut microbiota member belonging to this phylum, does not include homologues to *mlaA* nor *mlaC*, but it has an extended *mlaD*. AlphaFold2 predicts a striking structure for this protein, with a hexamer similar to *E. coli* MlaD in the N-terminus region but with a C-terminal long tunnel pointing towards the OM (Figure 4C). The tunnel has an open groove running in a coiled fashion with a hydrophobic interior. This is again reminiscent of the Lpt system involved in the transport of LPS from the IM to the OM (reviewed in [17]) with the proteins or domains spanning across the periplasm adopting a taco-shaped groove with a hydrophobic interior to allocate the acyl chains of the LPS while its hydrophilic region is solvent-exposed. One can speculate that this extended MlaD protein could be working in a similar fashion, accommodating the acyl chains of the PLs inside the channel and the polar heads facing the periplasm. This transport would be more efficient than using a cargo protein such as MlaC, potentially favouring anterograde transport. On the other hand, a more efficient system to remove misplaced outer leaflet PLs (retrograde transport) could offer an evolutionary advantage to a bacterium such as *B. theta* in constant contact with bile acids. It is worth noting that *B. theta* also possesses several paralogues of the aforementioned AsmA-like proteins. The predicted MlaD structure has a positively charged C-terminal region that could interact directly with the polar headgroups of the inner leaflet PLs (suggesting anterograde transport), but the presence of a functional homologue to *E. coli* MlaA in *B. theta* (suggesting retrograde transport) cannot be excluded (Figure 4C). It even seems possible that the directionality of Mla transport could have evolved differently in different groups of bacteria, with some having retrograde and other anterograde transport.

Phospholipid trafficking between the IM and OM is the last ‘black box' in building and maintaining the OM. Much has been done in recent years and we are starting to understand the processes implicated in PL transport. It is clear that there are still many interesting questions to be solved to improve our understanding about the Gram-negative bacteria cell envelope and that they will lead to many more exciting discoveries in the coming years.

## Summary

- The OM of Gram-negative bacteria is highly asymmetric with phospholipids in the inner leaflet and lipopolysaccharide molecules in the outer leaflet. Maintaining that asymmetry is vital to avoid entry of noxious compounds.
- The Mla pathway contains proteins in the OM, periplasm, and IM and has been linked to the removal of PLs in the outer leaflet of the OM (retrograde transport).
- *E. coli* MlaA is a donut-shaped α-helical protein with a central pore and it is almost entirely embedded into the inner leaflet of the OM. This allows MlaA to have access only to the outer leaflet of the OM for accepting PLs.
- Some reports suggest that PLs traffic, instead of being retrograde, could be anterograde or both. Functional, structural and recent biochemical studies strongly favour the retrograde transport.
- All considered, retrograde transport by the Mla system is the most likely and physiologically relevant.

## Abbreviations

ABC: ATP-binding cassette
IM: inner membrane
LPS: lipopolysaccharide
MCE: mammalian cell entry
OM: outer membrane
OMPs: outer membrane proteins
PLs: phospholipids
TMDs: transmembrane domains
